# Hepatitis E virus seroprevalence in pregnant women in Jiangsu, China, and postpartum evolution during six years

**DOI:** 10.1186/s12879-015-1308-y

**Published:** 2015-12-09

**Authors:** Guangyu Gu, Hongyu Huang, Le Zhang, Yongchun Bi, Yali Hu, Yi-Hua Zhou

**Affiliations:** Department of Laboratory Medicine, Nanjing Drum Tower Hospital, Nanjing University Medical School, Nanjing, 210008 China; Jiangsu Key Laboratory for Molecular Medicine, Nanjing University Medical School, Nanjing, 210008 China; Department of Obstetrics and Gynecology, Nanjing Drum Tower Hospital, Nanjing University Medical School, Nanjing, 210008 China; Department of Infectious Diseases, Nanjing Drum Tower Hospital, Nanjing University Medical School, Nanjing, 210008 China

**Keywords:** Hepatitis E virus, Seroprevalence, Pregnant women, Postpartum evolution, Children

## Abstract

**Background:**

China is an endemic area for hepatitis E virus (HEV). The previous surveys of anti-HEV seroprevalence are cross-sectional. This study aimed to investigate the prevalence of infection among pregnant women and their children in Jiangsu, China, and to observe postpartum anti-HEV evolution.

**Methods:**

Sera from 497 women collected during pregnancy and 6-year postpartum and from their 497 children were screened for anti-HEV by ELISA and confirmed by Western blotting. HEV RNA was detected by reverse transcription-nested PCR.

**Results:**

Of the pregnant women, 3 (0.6 %) were anti-HEV IgM positive and 55 (11.1 %) were IgG positive. At 6-year postpartum, 18 anti-HEV IgG positive samples became negative and 18 others became IgG positive; the accumulated prevalence in this cohort of women was at least 14.7 % (73/497). Of the 497 children, the positive rates of anti-HEV IgM and IgG were 0.2 % and 0.4 %, respectively. None of the 18 children from mothers with anti-HEV IgG seroconversion was anti-HEV IgG positive.

**Conclusions:**

Our data indicate that the constant seroprevalence of anti-HEV IgG in adults may be resulted from the balance of negative seroconversion due to waning immunity and positive seroconversion due to novel infections, and the risk of intra-family transmission of HEV was low. The data also imply that cross-sectional seroepidemiological survey may underestimate the prevalence of HEV infection, due to the natural decay of pathogen-specific IgG.

## Background

Hepatitis E is an acute, self-resolving infectious disease caused by hepatitis E virus (HEV). It is responsible for more than 50 % of adult acute viral hepatitis cases in endemic countries and 1 % in non-endemic countries [1]. Although most of HEV infections are mild or subclinical, the infection in pregnant women is particularly severe in high endemic countries. It has been reported that a significant proportion of pregnant women with hepatitis E may progress to fulminant hepatitis during epidemics, especially in the third trimester, with a high mortality rate of 15–20 % [2]. On the other hand, although HEV is mainly transmitted by the fecal-oral route, epidemiological and clinical studies have suggested that vertical transmission of HEV may frequently happen in HEV infected pregnant women and lead to adverse fetal outcomes, such as miscarriage, stillbirth and neonatal death [3, 4]. Moreover, although scarcely documented, the available data suggest secondary transmission of HEV may occur among family members both in outbreaks and sporadic infections [5–7]. However, fetal outcomes and person-to-person transmission of HEV among household members in China have received limited attention.

Sero-epidemiological studies have showed that the overall seroprevalence of anti-HEV IgG in African and European pregnant women were 11.6–84.3 % and 3.6–29.3 %, respectively [8–10]. In addition, in non-endemic and endemic areas, recent HEV infections could also occur in pregnant women. The positive rate of anti-HEV IgM in pregnant women in Spain and Ghana was 0.67 % [9] and 18.5 % [11], respectively. China is an endemic area for HEV. The reported seroprevalence of HEV infection in Chinese pregnant women was 10.24–16.2 % for anti-HEV IgG and was 2.56–3.2 % for anti-HEV IgM [12, 13]. However, these are cross-sectional studies. In addition, unlike infection with hepatitis A virus (HAV), it is reported that anti-HEV IgG antibodies only persist for months to years after infection [14] and it is meaningful to study the spontaneous decay of anti-HEV IgG. In the present study, we investigated the prevalence and potential risk factors of HEV infection among pregnant women in Jiangsu Province, and observed anti-HEV evolution in these postpartum women and their children after a 6-year follow-up. In addition, we also observed maternal and fetal outcomes during pregnancy in HEV infected individuals, and explored the possibility of intrafamilial HEV transmission.

## Methods

### Blood samples

Serum samples of 19,904 pregnant women, between 15 and 20 weeks of gestation, were collected from a project on the prevalence of Jiangsu Provincial Birth Defects in 14 cities or rural countries of Jiangsu Province from August 2002 to July 2004 by a multi-stage stratified cluster sampling method; these women was selected to be the representatives of the pregnant women population in Jiangsu, which had been designed by a specialist in medical statistics [15]. At a follow-up conducted from October 2009 to March 2010, serum samples from 497 paired mothers and their children were collected. Therefore, we evaluated the prevalence of anti-HEV in the 497 paired serum samples of pregnant women, mothers, and the children. All serum samples were stored at −30 °C until analyzed. The mean (± SD) age of 497 women was 25.0 ± 3.0 years in their second trimester and was 31.7 ± 3.5 years in the average at the follow-up conducted 6 years postpartum. For the 497 children, the mean age was 6.0 ± 0.6.

### Ethical statement

This study was approved by the institutional review boards (IRB) of Nanjing Drum Tower Hospital. In the retrospective analysis, each pregnant woman signed the written informed consent for herself and her child to participate in the birth defect study conducted from August 2002 to July 2004, and their serum samples were used in this study via an exemption approved by the IRB of Nanjing Drum Tower Hospital. In the follow-up study from October 2009 through March 2010, each mother gave the written informed consent for herself and her child for blood sampling (~3 ml) by venepuncture and for the use of her clinical records in the present investigation.

### Detection of anti-HEV

Anti-HEV antibodies were detected by several assays. Previously, we developed a unique ELISA to detect antibodies against HEV based on the combination of high reactivity of the recombinant immunodominant polypeptide and poor reactivity of the truncated polypeptide [16]. The recombinant immunodominant polypeptide, which contains the most immunogenic site on open reading frame 2 (ORF2) [17, 18], is derived from ORF2 of genotype 4 HEV, covering amino acids (aa 459–607), and the truncated polypeptide covers aa 472–607 [16]. In the present study, we used this ELISA as a primary screening assay to detect anti-HEV IgM and IgG respectively. The protocol was described previously [16]. The result was presented as optical density (OD_450_), which was read at 450 nm using a microplate reader (Bio-Rad, CA, USA). The OD_450_ value of the immunodominant polypeptide (aa 459–607) was denoted as OD_459–607_, and the truncated polypeptide (aa 472–607) was as OD_472–607_. Samples with OD_459–607_ ≥ 0.5 and OD_459–607_ : OD_472–607_ > 2 or OD_459–607_ – OD_472–607_ > 0.5, were considered to be positive for anti-HEV IgM or IgG.

Serum samples with positive results in the ELISA were further confirmed with Western blotting, which was based on the immunodominant polypeptide (aa 459–607). Our previous study has demonstrated that the antigenicity of polypeptide 459–607 is mainly depended on its dimeric characterization, since anti-HEV positive sera react only with the dimer, not with the monomer [16]. In sodium dodecyl sulphate-polyacrylamide gel electrophoresis (SDS-PAGE), polypeptide 459–607 presented only monomers when it was heated, and presented both monomers and dimmers when not heated [16]. Therefore, polypeptide 459–607 in Laemmli buffer without heating was used for Western blotting. In brief, the recombinant ORF2 polypeptide 459–607 was firstly separated by 12 % SDS-PAGE and then transferred to PVDF membranes. After blocked with 5 % skimmed milk in 0.05 % Tween-20 in phosphate-buffered saline at room temperature for 1 h, the membranes were incubated with serum samples diluted 20-fold in 5 % skimmed milk for 3 h. After three washes, HRP-conjugated anti-human IgM (1:5,000) or IgG (1:7,500) was incubated with the membranes for another 1 h. Anti-HEV IgM or IgG was detected by chemiluminescence using a Western blotting Luminol Reagent kit (Santa Cruz, CA, USA). A serum was finally interpreted as positive, when it was reactive to the dimeric form, but not to the monomer, of ORF2 polypeptide 459–607 in Western blotting.

Additionally, anti-HEV IgM positive samples were also tested by Wantai immunoassay (Wantai Biological Pharmacy Enterprize, Beijing, China), since the Wantai reagent for anti-HEV IgM has good specificity [19]. All steps and judgment criterion were performed in accordance with the manufacturer’s instructions.

### Detection of HEV RNA

Anti-HEV IgM positive serum samples were tested for HEV RNA using reverse transcription-nested PCR (RT-nested PCR), as described previously [16]. Total RNA was extracted from 200 μl serum using Trizol Reagent (Invitrogen, Carlsbad, USA). cDNA was then synthesized with a reverse transcriptase kit (TaKaRa, Tokyo, Japan). The ORF2 fragment was amplified using the primers U1stF (5’-CCGACAGAATTGATTTCGTC-3’), nt1150-1169 and U1stR (5’-TTACCYACCTTCATYTTAAG-3’), nt 1970–1951 as the outer pair; using the primers U2stF (5’-AAYGCTCAGCAGGAYAAGGG-3’), nt 1238–1257 and U2stR (5’-CAYTCHGGGCARAARTCATC-3’), nt1866-1847 as the inner pairs. The PCR conditions for both rounds were as follows: 94 °C for 5 min, 35 cycles at 94 °C for 30 s, 55 °C for 30 s, and 72 °C for 1 min, and a final extension at 72 °C for 5 min. Amplicons were separated by 1.5 % agarose gel electrophoresis and visualized by ethidium bromide fluorescence under the UV lamp.

### Statistical analysis

Statistical analysis was conducted using SPSS 13.0 (SPSS, Inc., Chicago, IL, USA). Data were presented as the mean ± SD. Chi-square test was used to compare anti-HEV seroprevalence in respect of age and residential area between groups. P ≤ 0.05 was considered to be statistically significant.

## Results

### Prevalence of anti-HEV in pregnant women and 6-year postpartum follow-up

Of the 497 pregnant women, 69 were positive for anti-HEV IgG and 6 were positive for anti-HEV IgM in the ELISA. In Western blotting, however, only 55 IgG positive and 3 IgM positive samples detected by the ELISA were finally confirmed to be reacted with dimeric ORF2 polypeptide and the remaining samples showed no reactions, indicating these were false-positive. Thus, the established positive rates of anti-HEV IgG and IgM in the pregnant women were 11.1 % (55/497) and 0.6 % (3/497), respectively. The three anti-HEV IgM positive serum samples, with normal ALT, were also positive for anti-HEV IgG. Factors associated with HEV infection of the pregnant women are shown in Table 1.

**Table 1:** Factors associated with HEV infection in pregnant women (*n* = 497) in Jiangsu, China

Variable	Category	Number of subjects tested	Number of positive subjects	%	*P* value
Age (years)	≤25	214	17	7.9	0.14
	26–30	235	34	14.5	
	31–35	40	3	7.5	
	>35	8	1	12.5	
Residential area	Urban	138	16	11.6	0.82
	Rural	359	39	10.9	

At the follow-up conducted at 6 years postpartum, 18 of the 55 anti-HEV IgG positive pregnant women turned to be anti-HEV IgG seronegative, and 2 of the 3 anti-HEV IgM positive became anti-HEV IgM negative (one was also converted into IgG negative). Meanwhile, of the remaining 442 anti-HEV IgG negative women during pregnancy, 18 seroconverted to anti-HEV IgG positive at the follow-up. As a result, there were still 55 (11.1 %) anti-HEV IgG positive women at 6 years postpartum. Since we tested the seroprevalence of anti-HEV IgG at two time points in the same population and the women who converted from anti-HEV IgG positive to negative should be considered to have been infected with HEV, the accumulated positive rate of anti-HEV IgG during the 6-year follow-up period was at least 14.7 % (73/497). Of the remaining 494 anti-HEV IgM negative individuals, two showed the IgM positive at the follow-up. Therefore, the positive rate of anti-HEV IgM was still 0.6 % (3/497) in these women. The serum ALT levels and clinical status of those anti-HEV positive women were all normal. It was noteworthy that one sample, which was positive for both anti-HEV IgM and IgG in the second trimester, was still positive for both anti-HEV IgM and IgG at the follow-up.

### Prevalence of anti-HEV in children

A total of 497 children, 280 boys and 217 girls, were included in the study. Their average age was 6.0 ± 0.6 years. The prevalence of anti-HEV IgG in children was 0.4 % (2/497), significantly lower than that in their mothers (*χ*^*2*^ = 52.28, *p* <0.05). Among them, only one (1/497, 0.02 %) was anti-HEV IgM positive, with anti-HEV IgG positive simultaneously. These two children had no symptoms and signs of hepatitis and had no elevation of ALT. They were both girls; one’s mother was positive for anti-HEV IgG at pregnancy but converted to negative at follow-up, and the other’s mother was anti-HEV negative at both pregnancy and follow-up.

In addition, all available anti-HEV IgM positive serum samples were also shown positive results after retested by Wantai ELISA kit (Table 2). In the three anti-HEV IgM positive pregnant women, no adverse pregnant and neonatal outcomes were observed and their children were all anti-HEV seronegative. None of the 18 children whose mothers underwent postpartum anti-HEV seroconversion was anti-HEV positive.

**Table 2:** Comparison of anti-HEV IgM with in-house ELISA and Wantai ELISA

Sample	In-house ELISA			Wantai ELISA	
	OD_450_		Result^a^	OD_450_	Result^b^
	Polypeptide	Polypeptide			
	459–607	472–607			
B024042	1.105	0.409	+	0.294	+
F430527	1.377	0.429	+	0.403	+
193A	1.142	0.437	+	0.309	+
315A	1.392	0.502	+	0.309	+

^a^ELISA microplates were separately coated with purified immunodominant recombinant ORF2 polypeptide 459–607, and the truncated polypeptide 472–607. The OD_450_ value of the polypeptide 459–607 was denoted as OD_459–607_, and that of the polypeptide 472–607 was as OD_472–607_. Samples which showed OD_459–607_ ≥ 0.5 and the ratio of OD_459–607_ and OD_472–607_ > 2 or had the difference of OD_459–607_ minus OD_472–607_ > 0.5 were considered to be positive for anti-HEV IgM

^b^The OD_450_ values of 0.263 or above were considered to be anti-HEV IgM positive according to the instruction of the Wantai ELISA

### Detection of HEV RNA

Four serum samples with anti-HEV IgM positive were available for further testing HEV RNA, including two obtained from pregnant women and two from 6-year postpartum follow-up mothers. All tested serum samples were also positive for anti-HEV IgG simultaneously, along with normal ALT levels. However, none of the tested serum samples were positive for HEV RNA.

## Discussion

In the present study, we detected anti-HEV IgM and IgG in a cohort of 497 women at mid-term pregnancy and at 6 years postpartum and also in their 497 children. We found that the positive rate of anti-HEV IgM or the prevalence of anti-HEV IgG was constant in women at the two time points. The unchanged positive rates do not reflect that there was no novel HEV infection occurred during the observation period, but it was resulted from the balance of seronegative conversion due to the natural decay of anti-HEV IgG and the seropositive conversion caused by the novel infections. Additionally, we found that anti-HEV prevalence in Chinese children was extremely low.

Previous studies have indicated that commercially available immunoassays for detection of anti-HEV antibodies differ dramatically in the sensitivity and specificity. The sensitivity could range from 72 to 98 % in detecting the same diagnostic sensitivity panel [20], and highly discrepant results existed in evaluation of anti-HEV IgG seroprevalence by different assays in the same serum panel, ranging from 4.5 to 29.5 % [21]. In recent years, antigen derived from ORF2 polypeptide of genotype 4 has been proved to display good diagnostics performance in detecting antibodies against genotypes 1, 3, and 4 of HEV [22–24]. Since HEV genotype 4 is dominant in China [25], we considered that our ELISA based on the ORF2 of genotype 4 is appropriate to detect anti-HEV in China; the ELISA may largely exclude false-positive results caused by nonspecific binding based on the combination of high reactivity of purified immunodominant ORF2 polypeptide 459–607 and poor reactivity of the truncated polypeptide 472–607 [16]. Moreover, all anti-HEV positive serum samples in the ELISA were verified by Western blotting, which further reduced the likelihood of false-positive results caused by ELISA. In addition, as an approach for testing the reliability of our assays, all anti-HEV IgM positive samples were also tested to be positive by the highly specific Wantai anti-HEV IgM ELISA kit, indicating good specificity of our two combination assays. Thus, our results would be more reliable to reflect the real situations of HEV infection.

Our results showed that the prevalence of anti-HEV IgG in the second trimester of pregnant women in Jiangsu province was 11.1 %, relatively lower than that (15.5 %) in the pregnant women in Shandong province [12]. In addition, the positive rates of anti-HEV IgM in the mid-term pregnant women and 6-year follow-up were each 0.6 %, lower than these recently reported in Shandong province (3.2 %) [12] and Yunnan province (2.56 %) [13]. The differences in the positive rates of anti-HEV IgG or IgM in different areas of other countries were also documented in the literature. For example, in Japanese blood donors with elevated ALT levels, by detecting anti-HEV IgG with different ELISAs, the positive rates in Honshu and Hokkaido were 1.7 % and 3.2 %, respectively [26, 27]. One may assume that the variations in the positive rates may reflect the real situations in different areas of the same country. However, except for the factors of different sex and age structure and sampling errors, we considered that the different antigens used in the ELISA assays of detecting anti-HEV IgG may be a more important reason. Fukuda et al. [26] applied antigen derived from ORF2 protein of HEV genotype 4 and observed a relatively low positive rate, whereas Sakata et al. [27] applied antigen derived from ORF2 protein of HEV genotype 1 and with a high positive rate. Thus, the relatively lower prevalence of anti-HEV IgG in our study may be attributed to the more specific assay used in the present study. Additionally, unlike previous studies [12, 28], we did not find significant associations of age and type of residential area (urban/rural) with the prevalence of anti-HEV. It is hard to explain the reason at this time.

Recently, several studies have surveyed changes in anti-HEV IgG prevalence over time [29–31]. However, all those studies enrolled different populations at various time points, leaving the comparison less meaningful. In our present study, through a cross-sectional and a 6-year interval follow-up study in the same population, we observed that the prevalence of anti-HEV IgG was dynamically constant. This is in agreement the notion that the prevalence of anti-HEV IgG remains essentially stable after people reaching a certain age [32]. Additionally, during the 6-year postpartum follow-up, we observed that 18 women with positive anti-HEV IgG during pregnancy became seronegative, which is conforming the phenomenon of spontaneous decay of anti-HEV IgG antibodies in the years after infection [14]. According to this serological feature of HEV, we suppose that the constant prevalence of anti-HEV IgG in adults may be resulted from the balance of natural decay of anti-HEV IgG and seroconversion to anti-HEV IgG due to incidental infection. Moreover, for those who were persistently positive for anti-HEV IgG during the pregnancy and at the follow-up, we could not clarify whether it was due to the long lasting of anti-HEV IgG or due to the re-infection in these women since re-infection of HEV may occur in the presence of anti-HEV [33].

Our results based on the longitudinal observation in the same cohort of women imply that cross-sectional survey may underestimate the prevalence of anti-HEV IgG. Based on the cross-sectional surveys, the positive rate of anti-HEV IgG either during pregnancy or postpartum happened to be equal to 11.1 %. However, the accumulated prevalence at 6 years postpartum should be at least 14.7 %, since we just tested anti-HEV at an interval of 6 years and could not identify the possible cases of anti-HEV seroconversion which then turned seronegative in the study period. It was also reasonable to speculate that some of the women with negative anti-HEV during the mid-trimester actually had past HEV infections and subsequently became seronegative.

Generally, children are especially vulnerable to infectious diseases transmitted via the fecal-oral route. For example, HAV infection usually occurs before the preschool age, reaching a peak at about 6 years old [34]. HEV, which is also an enterically transmitted virus, however, the infection rate in children is low when compared with adults. For example, the seroprevalence of anti-HEV IgG increased from 1 % in 0–17 years to 18 % in German adults [21, 35]. In our study, the prevalence rate of anti-HEV IgG in children was 0.4 %, also significantly lower than that in their mothers. We assume that one possible reason is that, compared with the adults, children have extremely low chances to expose to the virus, although they are susceptible to enterically transmitted hepatitis. On the other hand, the reported prevalence of anti-HEV IgG by Jia et al. [36] in 1–14 years old Chinese children was 6.77 %, which is much higher than our results. The different laboratory methods, just like mentioned above, may be responsible for the variations. Thus, the prevalence of anti-HEV IgG in children merits further investigation by high quality reagents or by several assays in parallel. In addition, person-to-person transmission of HEV has been reported and secondary attack rate among household transmission of HEV was ranging from 0.7 to 2.0 % [5–7]. In our study, none of the 18 children whose mothers experienced postpartum anti-HEV IgG seroconversion was anti-HEV positive, indicating the low risk of intra-family HEV infection.

In the present study, all anti-HEV IgM positive individuals were in normal ALT levels. However, since we did not examine the pregnant women at regular intervals and ALT is not always elevated in acute HEV infections [37], we could not figure out whether they had subclinical infections or symptomatic hepatitis E before the normalization of ALT levels. In addition, none of tested anti-HEV IgM positive serum samples had detectable HEV RNA. Excluding the low detection efficiency of RT-nested PCR, the transient existence of viremia [38] or low viral load [39] in these patients may be the main reasons. Additionally, studies have demonstrated that HEV infection in pregnant women can result in intrauterine growth restriction, abortions, still births or neonatal death [3, 4]. In the present study, we did not observe any adverse effects on the pregnant and neonatal outcomes in the three pregnant women with new infections. It may be due to the small number of cases or due to the possibility of less severe conditions of these pregnant women.

One limitation in the present study is that we did not comprehensively evaluate the risk factors for infection of HEV in the study subjects, leaving the possible reasons for the novel infections happened in postpartum period unresolved. Second, one woman who was positive for both anti-HEV IgM and IgG over 6 years had normal ALT levels and had no detectable HEV RNA. Whether she was chronically infected or had unusual profile of anti-HEV response or had the possibility of repeated infection remains to be elusive. Third, the lack of HEV RNA detection makes it impossible to analyze the genotypes of sporadic HEV infections in Jiangsu, China.

## Conclusions

The current study is useful to understand the prevalence of HEV infection in pregnant women in China. The data from the longitudinal observation in pregnancy and postpartum indicate that the constant prevalence of anti-HEV IgG in adults may be resulted from the balance of negative seroconversion due to waning immunity and positive seroconversion due to novel HEV infections. Our data also imply that cross-sectional seroepidemiological survey may underestimate the prevalence of HEV infection and possibly other pathogenic infections, due to the natural decay of pathogen-specific IgG. Person-to-person transmission of HEV is an uncommon event.
